# The *Endocrine Feedback Loop* Podcast: Past, Present, and Future

**DOI:** 10.1210/jendso/bvae183

**Published:** 2024-10-23

**Authors:** Chase D Hendrickson

**Affiliations:** Division of Diabetes, Endocrinology, and Metabolism, Department of Medicine, Vanderbilt University Medical Center, Nashville, TN 37212, USA

**Keywords:** podcast, journal club, medical education

## Abstract

In June 2024, the *Endocrine Feedback Loop* podcast recorded its fiftieth episode at the annual meeting of the Endocrine Society. Launched in May 2020, the podcast serves as a way for listeners to hear a critical analysis of recent and impactful research studies published in clinical journals of the Endocrine Society. The podcast follows methods proven effective in traditional journal clubs and adapts them to reach a wide audience with different levels of expertise in endocrinology. This *Perspective* outlines the history of the podcast, the process of producing a monthly episode, and the topics covered, as well as an assessment of the experience of producing the podcast and future plans for the *Endocrine Feedback Loop*.

For nearly 200 years, journal clubs have served an important role for physicians. Journal clubs started as a means for physicians to meet and discuss the most recent research, keeping them up to date on the medical literature. They later evolved to introduce peer-to-peer teaching into traditional medicine. More recently, journal clubs have helped teach trainees critical appraisal of biomedical research [1]. Podcasts entered into the field of medical education several decades ago. A recent review of the effect of podcasts in medical education reported that podcasts appeal to audiences at all stages (students, trainees, and independent physicians), increase listener knowledge, and lead to a reported change in clinical practice [2]. Physicians include podcasts when reporting their preferred methods for staying current with the medical literature [3]. In recent years, medical-education podcasts styled as journal clubs have launched, attempting to marry a traditional, interactive discussion to a modern, one-way delivery method. Such an approach demonstrated effectiveness in teaching evidence-based medicine, based on self-reporting by residents [4].

In 2019, the Endocrine Society started laying plans to launch its own journal club podcast, the *Endocrine Feedback Loop*. While reminiscent of traditional journal clubs based within training programs, this podcast was designed to focus on conversations between faculty members at endocrinology programs. The podcast would review clinical research published in one of the Endocrine Society's clinical journals, *The Journal of Clinical Endocrinology & Metabolism* (*JCEM*) and the *Journal of the Endocrine Society* (*JES*), focusing on recently published articles covering the breadth of the field. The podcast Host invited 12 individuals to participate in the role of Regular Contributor for the first 3 years. These individuals were chosen based on experience in endocrine education at academic institutions throughout the United States, with a focus on program directors and associate program directors of endocrinology fellowships. The programs chosen represented different geographic regions throughout the United States and included a pediatric program.

Though essentially consisting of recorded episodes, the *Endocrine Feedback Loop* podcast was designed to contain elements shown to make traditional journal clubs successful [5]. As examples, each episode includes only endocrinologists, keeping discussions focused on aspects relevant to those in the field. The participants prioritize critical analysis of the study, with the use of standard assessment tools to aid in the analysis process. Each episode follows a consistent pattern, with a single Host leading the discussion and highlighting key methodological and statistical aspects. Only clinical research articles are chosen. Each episode ends with placing the findings into the context of clinical practice, with a discussion of whether the results should change current clinical practice.

## Production Cycle

For each episode of the *Endocrine Feedback Loop* podcast, the production cycle follows a consistent pattern of article selection, Guest Expert identification, article analysis, discussion guide creation, and recording. While the method of article selection evolved over the first year of the podcast (discussed below), the rest of the process has remained quite stable.

### Article Selection and Guest Expert Identification

To begin the production cycle, the podcast Host reviews recent clinical research articles from *JCEM* and *JES*, both those published in their final form and those published as “Advance Articles” (accepted articles awaiting copyediting). The Host evaluates each article based on relevance to a broad audience of endocrinologists, focusing on studies with practice-changing implications, novel conclusions, and topics currently of high interest in the field. Initially, articles were chosen regardless of any subspecialty expertise held by the Regular Contributor for that episode. As it became evident that more nuanced discussions were held between the Regular Contributor and Guest Expert when both brought subject-matter expertise to the topic, the podcast introduced another selection criterion: for Regular Contributors with a specific area of expertise, the article would typically be within their field. Once the Host and Regular Contributor agree upon an article, the Regular Contributor suggests a Guest Expert to invite to participate in the episode. Guest Experts are chosen based on recognized authority on a specific topic and cannot be authors of the article being reviewed.

### Article Analysis

Once an article is identified, the Host and the Regular Contributor separately critically appraise the entire article using structured review instruments. These structured review instruments, created for specific study designs, identify weaknesses innate to each study design, especially biases [6]. The Host and the Regular Contributor each note areas of concern/disagreement with the authors and identify areas where an expanded discussion would be of interest to the audience or provide important clinical implications.

### Discussion Guide Creation

After the analysis is complete, the Regular Contributor drafts a discussion guide. The guide follows the standard flow of a research article (Introduction, Methods, Results, Discussion), with each section receiving roughly equal content. In the Introduction section, questions are included that will be posed to the Guest Expert, typically requesting an expanded discussion on important pathophysiologic, diagnostic, or therapeutic concepts. The Methods section starts with an overview of the study design utilized by the authors. Pertinent aspects of the study design are reviewed, with questions to the Guest Expert as deemed helpful. While the Introduction and Methods portions tend to be comprehensive, the Results section utilizes a focused review of the data. This selectivity aims to avoid “information overload,” prioritizing the verbal description of complex numerical findings without the aid of accompanying visuals. The Discussion section details the authors' summary of their findings. It includes questions to the Guest Expert when the analysis identified statements potentially not supported by the data. The guide undergoes multiple rounds of edits between the Host and Regular Contributor and is then sent to the Guest Expert for additional input.

### Recording

The podcast recording usually occurs via teleconference, with the exception of an in-person recording held during the annual meeting of the Endocrine Society. An episode length of 40 to 45 minutes is targeted. The Host starts the recording with a scripted introduction of the topic, the participants, and the article. The focus then shifts to the prepared guide, utilizing it as an outline and not an exact script in order to maintain a conversational tone throughout the duration of the recording. The Regular Contributor and Host alternate leading the conversation through the 4 sections of the paper. While discussing the study's methodology, the Host focuses on the study design as the key to understanding the critical analysis of the article. The podcast ends with a discussion of the overall quality of the study and a question of whether the article's findings should change the practice of clinical endocrinology or if more research is needed. For the recordings that occurred at the 2023 and 2024 ENDO meetings, 4 endocrinology fellows joined each episode. They helped to analyze the article in advance and asked prepared questions of the Guest Expert. The production team with the Endocrine Society makes post-recording edits prior to releasing each episode.

## By the Numbers

Between May 2020 and June 2024, the podcast produced 50 monthly episodes. Table 1 summarizes the topics of the articles reviewed, with diabetes mellitus being the most frequently covered topic (12 episodes) and obesity being the least (4 episodes). Throughout these first few years, several volunteers rotated out of the role of Regular Contributor and were replaced. A total of 18 Regular Contributors participated in these sessions, comprised of 17 adult endocrinologists and 1 pediatric endocrinologist. A total of 49 Guest Experts joined these episodes, including 44 adult endocrinologists and 5 pediatric endocrinologists. Of the Guest Experts, 42 came from institutions within the United States, and 7 hailed from international ones (3 in Europe, 2 in Australia, 1 in South America, and 1 in Asia). For each year of the podcast, 1 article was chosen from the *JES*, with the rest coming from the *JCEM*.

**Table 1.** A summary of topic areas covered in the first 50 episodes of the *Endocrine Feedback Loop* podcast

Topic area	Number of articles
Adrenal	7
Bone/Calcium	7
Diabetes Mellitus	12
Obesity	4
Pituitary	6
Reproductive	9
Thyroid	5

Given the initial method of hosting the podcast internally (see below), only rudimentary user data can be presented. Over 10 000 users visited pages on the podcast website, with visits ranging from over 800 for the most popular episode to the 50s for the least popular episodes. Numbers of actual downloads and listens cannot be determined. No patterns related to topic area and site visits are apparent. Of note, the podcast launched in the early days of the COVID-19 pandemic, although it had been planned for many months in advance and so was coincidental to an event that may have encouraged listening.

## Lessons Learned and Future Directions

### Accessibility

For the first 4 years of the podcast, the recordings could be accessed only via an Endocrine Society membership through the Endocrine Society website. This method of hosting the podcast effectively demonstrated the benefits of a membership in the Society. However, doing so significantly limited its visibility and increased the difficulty of accessing and downloading each episode. This limitation potentially illustrates why such podcasts are not typically produced by professional societies [7]. Upon starting the fifth year of the podcast, the podcast moved to be freely available on multiple platforms (eg, Spotify, Apple, YouTube). While too early to assess the impact, the move makes access far easier, hopefully leading to an increased listenership and fostering future growth of the podcast. Listens can now be tracked, allowing for a true quantification of the audience and identification of the factors making some episodes more popular than others. Ongoing promotion by the Endocrine Society will highlight the work the Society does for the field while helping the podcast overcome the usual reliance on “word of mouth” to grow and reach a large, global audience [2].

### Expertise of Regular Contributors

The quality of the discussion during episodes increased substantially when Regular Contributors with a subspecialty focus reviewed articles within their area of expertise. Such episodes became more in-depth peer conversations between the Regular Contributor and the Guest Expert, with the Host focusing on facilitating the conversation and providing input on the methodology employed in the study. Conversely, Regular Contributors who are general endocrinologists brought substantial breadth to their episodes, allowing for the selection of articles of high interest to listeners without the need to match it to a specific area of expertise. Additionally, these general endocrinologists helped the Host focus on the educational content and apply these findings to a broad audience. Thus, the podcast will continue with a mix of Regular Contributors who are general endocrinologists and who are subspecialists with diverse areas of focus.

### Enhancing Engagement and Impact

As a one-way method of content delivery, the *Endocrine Feedback Loop* podcast does not naturally engage listeners beyond passive reception. Traditional journal clubs focus on participation of listeners, effectively replicated in some online journal clubs [1]. The podcast did facilitate listener engagement by trialing audience participation in selecting an article for discussion via a survey on social media, with good participation and the choosing of an article based on that input. Creating additional opportunities for direct involvement of listeners will help overcome the natural limitations of the podcast medium. Such input could be used to regularly guide article/topic selection and even the questions posed to the Guest Expert. With the podcast now hosted on widely available platforms, listeners can now leave feedback via those platforms. As another recent change, each episode now ends with a mention of the email address through which listeners can also provide input.

To improve the impact of the *Endocrine Feedback Loop* podcast, the podcast needs to better understand the needs of its listeners. The podcast hopes to reach listeners ranging from medical residents and endocrinology fellows in the earliest stages of their careers to the most experienced endocrinologists. Training programs may be able to provide insight into how the podcast can aid in their educational efforts. Additional input from practicing endocrinologists will help determine if the podcast effectively keeps them up to date on the medical literature while reviewing fundamentals of endocrinology and teaching critical appraisal of the medical literature. The loftiest goal of the podcast would be to improve the endocrine care that its listeners provide to their patients.

## Conclusion

The *Endocrine Feedback Loop* podcast launched more than 4 years ago and serves as a way for endocrinologists worldwide to listen to experts in the field present their assessment of recently published, clinically relevant endocrine research. Listeners go from an independent reading of an article in an Endocrine Society journal to hearing a dynamic conversation critically analyzing important research. Occurring monthly, the podcast gives endocrinologists a practical way to get immediate insight from experts as to whether their practice should change, far in advance of meetings of professional societies or updates of clinical guidelines. With a move to be widely available, the podcast hopes to reach a larger audience in an increasingly impactful fashion.

## Data Availability

Some or all datasets generated during and/or analyzed during the current study are not publicly available but are available from the corresponding author on reasonable request.
